# Differentiation Between Organic and Non-Organic Apples Using Diffraction Grating and Image Processing—A Cost-Effective Approach

**DOI:** 10.3390/s18061667

**Published:** 2018-05-23

**Authors:** Nanfeng Jiang, Weiran Song, Hui Wang, Gongde Guo, Yuanyuan Liu

**Affiliations:** 1 Digit Fujian Internet-of-Things Laboratory of Environmental Monitoring, School of Mathematics and Informatics, Fujian Normal University, Fuzhou 350007, China; jiangbbplayer@163.com (N.J.); 15705957029@163.com (Y.L.); 2 School of Computing, Ulster University, Belfast, BT37 0QB, UK; Song-W@ulster.ac.uk (W.S.); h.wang@ulster.ac.uk (H.W.)

**Keywords:** sensor system, diffraction grating, computer vision, pattern recognition, organic apple

## Abstract

As the expectation for higher quality of life increases, consumers have higher demands for quality food. Food authentication is the technical means of ensuring food is what it says it is. A popular approach to food authentication is based on spectroscopy, which has been widely used for identifying and quantifying the chemical components of an object. This approach is non-destructive and effective but expensive. This paper presents a computer vision-based sensor system for food authentication, i.e., differentiating organic from non-organic apples. This sensor system consists of low-cost hardware and pattern recognition software. We use a flashlight to illuminate apples and capture their images through a diffraction grating. These diffraction images are then converted into a data matrix for classification by pattern recognition algorithms, including *k*-nearest neighbors (*k*-NN), support vector machine (SVM) and three partial least squares discriminant analysis (PLS-DA)- based methods. We carry out experiments on a reasonable collection of apple samples and employ a proper pre-processing, resulting in a highest classification accuracy of 94%. Our studies conclude that this sensor system has the potential to provide a viable solution to empower consumers in food authentication.

## 1. Introduction

Food authentication is a process to analyze the composition of food to ensure the food is “what it says on the tin”. It is increasingly needed in many areas of science, agriculture and business for various reasons, including the growing demand for high-quality food products. Classical chemistry analyzes materials through chemical reactions that mostly occur in the liquid phase, which is generally expensive, time consuming and requires professional laboratory techniques for food authentication. This approach is therefore unsuitable for routine authentication in consumer markets and unable to effectively control food fraud, such as organic food mislabeling and mixing.

In last decade, there has been a growing trend towards fast and non-destructive approaches for food authentication. A popular approach is to differentiate one food type from another by using spectroscopic techniques such as near-infrared (NIR), Fourier-transform infrared (FTIR) and nuclear magnetic resonance (NMR) with the aid of chemometrics (chemical pattern recognition) [1,2,3]. It studies the interaction between material and electromagnetic radiation as a function of light intensity over wavelength or frequency. Then the specific chemical compositions or physical properties of the material can be revealed by means of chemometrics analysis. This approach has been investigated in many food quality studies, including identifying varieties [4], pesticide residues [5] and transgenics [6]. However, to our knowledge, such an approach is seldomly used to detect organic products which have higher value than non-organic ones, but are less distinctive in surface morphology and easier to adulterate. Recent studies attempted to use portable NIR spectroscopy for differentiating organic foods from non-organic ones, such as apples [7,8] and milk [9]. These studies have demonstrated that portable spectroscopy together with chemometrics is a potential alternative to laboratory-based spectroscopy due to its classification efficiency. Nevertheless, this alternative method remains difficult to apply in consumer markets. On one hand, the miniaturization and field portability of instrumentation generally lowers the fingerprint data quality due to the non-ideal sampling conditions such as stray light, detector-based and chemical-based effects [10]. As a result, nonlinear variations might be introduced into spectral data, degrading the performance of linear classification models [11,12]. On the other hand, the cost of using a portable spectroscopic sensor for differentiating organic food drastically exceeds the expectation of consumers. For example, the portable NIR spectrometer used in [7] costs over ten thousand British pounds while the price of a smaller-sized SCIO spectrometer [13] is still almost 300 US dollars. Therefore, low-cost sensors which can provide comparably effective performance to portable spectroscopy are desired and will have huge potentials in application for food authentication.

Many pattern recognition algorithms have been applied in chemometrics for discovering the connection between sensory food data and their corresponding chemical components or physical properties [1,14]. Among these algorithms, *k*-NN, SVM and PLS-DA have been frequently used as baseline methods due to their simplicity and easy implementation. Specifically, PLS-DA is practically suitable for modelling ill-conditioned problems in spectral data, such as small samples, high dimensionality and multicollinearity. However, it suffers from performance degradation under nonlinear conditions [11,15]. Recent studies attempt to modify PLS algorithm for handling nonlinear data by using kernel and locally weighted approaches [15,16]. Kernel PLS (KPLS) maps the original data into Hilbert feature space and constructs a linear PLS model. According to the Covers theorem, the nonlinear relationship among variables in the original data space becomes linear in the feature space after such mapping [17]. Therefore, KPLS can effectively capture the nonlinearity and improve the prediction performance. However, it is not possible to directly see the contribution of each variable with respect to the prediction model [15]. Furthermore, kernel methods are prone to overfitting if the dataset has a limited number of samples [11]. Another approach combines locally weighted regression (LWR) [18] and PLS, namely LW-PLS [16]. It fills the gap that the regression coefficients of LWR are unstable and inefficient to compute under ill conditions. LW-PLS is a Just-In-Time (JIT) method which builds a local regression model based on the distances between training samples and a query. This model can enlarge the contribution of neighboring samples for the query and reduce global nonlinearity. It has been reported that LW-PLS is superior to the conventional PLS in developing soft-sensors [19,20].

In this paper, we present a low-cost sensor system based on computer vision techniques for authentication purposes, i.e., differentiating organic apples from non-organic ones. This sensor system consists of simple components (the hardware costs less than 3 US dollars on top of a smartphone) which are consumer-friendly and do not require expert knowledge to operate. It aims to provide rapid and non-destructive analysis that can effectively reveal the relationship between food data and its categorical information. In particular, the apple data acquired by our sensor system exhibits strong nonlinearity when the categorical information is based on organic and non-organic. We use LW-PLS method to improve the modelling performance and achieve classification capability that is comparable to portable NIR spectrometers in differentiating organic apples from non-organic ones.

The remainder of the paper is organized as follows: Section 2 introduces the sensor system and image data acquisition process. The image data analysis is presented in Section 3. Section 4 presents the experimental results and discussion. Conclusions are drawn in Section 5. 

## 2. Measurements

### 2.1. Sensor System

**Figure 1:**
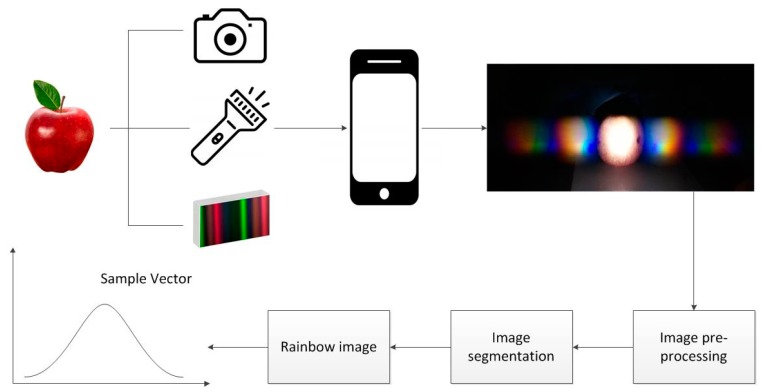
The overall sensor system for data acquisition.

The proposed sensor system aims to acquire image data from certain objects, i.e., organic and non-organic apples, by coupling low-cost measurements with computer vision techniques. Using a simple flashlight to illuminate an apple, a diffraction image is generated and captured by a diffraction grating sheet and camera, respectively. Then we apply a series of computer vision techniques, including image pre-processing, segmentation and rainbow generation to convert the diffraction image into a sample vector for analysis. The overall sensor system is shown in Figure 1.

### 2.2. Diffraction Grating and Image Acquisition

**Figure 2:**
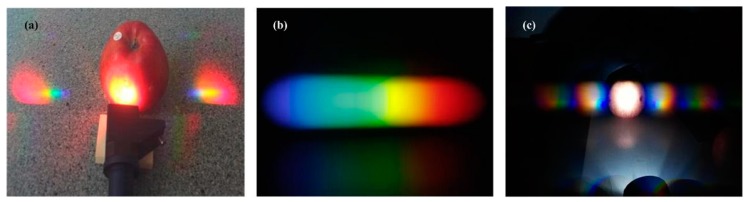
(**a**) Using flashlight to illuminate an apple sample; (**b**) the rainbow from the diffraction grating sheet; (**c**) the experimental condition and reflected rainbow images.

Diffraction gratings are an essential optical component in many fields such as spectroscopy, lasers techniques and optical communication. When polychromatic light reflects from or passes through a diffraction grating, it disperses into several rays travelling in different directions and each ray contains a unique wavelength or color. If we use a flashlight to illuminate an apple, spectra are generated on both sides of the apple which are severely dispersive (see Figure 2a). Diffraction grating can equidistantly slit a spectrum by wavelengths and produce rainbow color spectrum (also called rainbow, shown in Figure 2b) if a wide spectrum light source is being used. As different chemical elements (or compounds) have unique spectra which differ in the composition of the spectral lines, it is possible to detect the presence of a specific element by analyzing the intensity of a spectral line. In this paper, we choose a 60 × 40 nm diffraction grating sheet (less than 3 US dollars) as the experimental object. According to Figure 2c, rainbow images of an apple are generated via the diffraction grating sheet and their color spectra are symmetrically distributed.

To reduce the influence of ambient light and produce images of high quality, the experiment was conducted in a dark environment. There is no surface contamination or damage in each apple and no surface preparation was carried out prior to image acquisition. We place a flashlight 20 cm away from the apple and set diffraction grating sheet right behind the flashlight, which can ensure that the light source is effectively focused on the apple surface. After generating rainbow images, a smartphone is used to photograph the whole experimental environment.

### 2.3. Rainbow Images Segmentation

#### 2.3.1. The Proposed Framework

**Figure 3:**
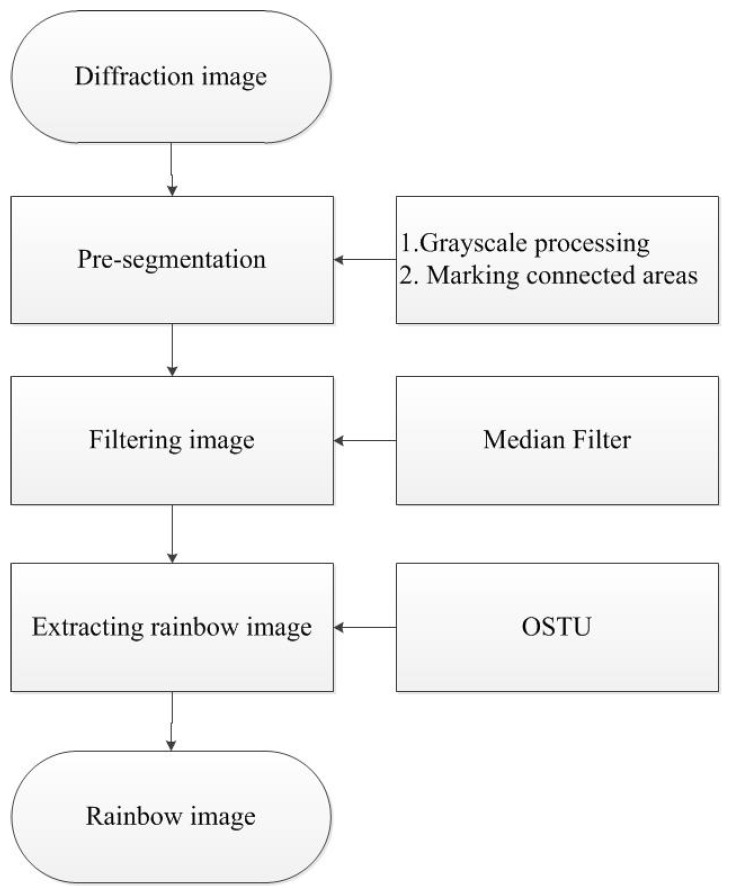
The proposed framework for extracting rainbow image.

**Figure 4:**
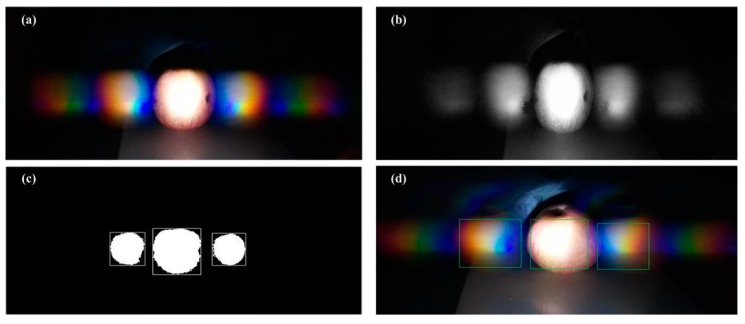
(**a**) The original image; (**b**) gray scale processed image; (**c**) binary image; (**d**) the resulted image.

To capture a single rainbow image, the background needs to be removed. Image segmentation is the process of detecting objects or interesting areas from input image which plays a key role in object recognition. We propose a framework which can efficiently extract one rainbow image, as shown in Figure 3. We use pre-segmentation techniques to pre-process the image, including grayscale processing and mathematical morphology. Then we apply median filter to denoise the image. By using OSTU (Nobuyuki Otsu) method [21], a single rainbow image is extracted from the original image and converted into color histogram vectors in RGB color space. Figure 4 shows the original and processed images by the above procedures.

#### 2.3.2. Image Denoising

Digital images are generally affected by noise from the imaging instrument and the external environment during digitization and transmission. In this paper, we use the median filter for image denoising. The main idea of median filtering is to replace the value of a point in a digital image or digital sequence with the median of neighboring points. As a result, the surrounding pixel values are close to their real value and the isolated noise points are eliminated. The median filtering is especially useful for denoising which can efficiently preserve edges [22]. After image denoising, to retain the maximum information of color, we used the hue, saturation and value (HSV) color model, which is close to human visualization for preliminary processing the rainbow image.

#### 2.3.3. The OSTU Method

The OSTU method [21] is an automatic image segmentation algorithm which find a threshold that minimizes the weighted within-class variance. In this paper, we use the OSTU method to distinguish a rainbow image from background due to its simplicity and effectiveness. It firstly converts input images to grayscale images and count the number of gray levels *K* (0 < *K* < 255). Then pixels are divided into background class *C*1 and object class *C*2 by a threshold *T*, *C*1 and *C*2 are within the interval of [1, *T*] and [*T* + 1, *K*], respectively. We define the total number of pixels as *L* and the probability of appearance grayscale level as *μ*, the algorithm is summarized as follows:

Calculate probability appears of *C*1:

(1) $$\omega_{1}\left(T\right)=P_{1}=\sum_{i=0}^{T-1}p_{i},$$

Calculate probability appears of *C*2:

(2) $$\omega_{2}\left(T\right)=P_{2}=\sum_{i=0}^{K-1}p_{i}=1-P_{1},$$

Calculate the average grey level of *C*1:

(3) $$\mu_{1}\left(T\right)=\sum_{i=0}^{T-1}i\cdot P\left(\frac{i}{C_{1}}\right)=\sum_{i=0}^{T-1}i\cdot p_{i}/\omega_{1}\left(T\right),$$

Calculate the average grey level of *C*2:

(4) $$\mu_{2}\left(T\right)=\sum_{i=0}^{L-1}i\cdot P\left(\frac{i}{C_{2}}\right)=\sum_{i=0}^{L-1}i\cdot p_{i}/\omega_{2}\left(T\right),$$

Calculate the sum of the average grey level:

(5) $$\mu =\omega_{1}\left(T\right)\cdot \mu_{1}\left(T\right)+\omega_{2}\left(T\right)\cdot \mu_{2}\left(T\right),$$

Calculate the interclass variance:

(6) $$g\left(T\right)=\omega_{1}\left(T\right)\cdot {\left(\mu_{1}\left(T\right)-\mu \right)}^{2}+\omega_{2}\left(T\right)\cdot {\left(\mu_{2}\left(T\right)-\mu \right)}^{2},$$

Calculate:

(7) $$g\left(T\right)=\omega_{1}\left(T\right)\cdot \omega_{2}\left(T\right)\cdot {\left(\mu_{2}\left(T\right)-\mu_{1}\left(T\right)\right)}^{2}.$$

The optimal threshold *T* can be obtained when *g*(*T*) achieves the maximum value.

**Figure 5:**
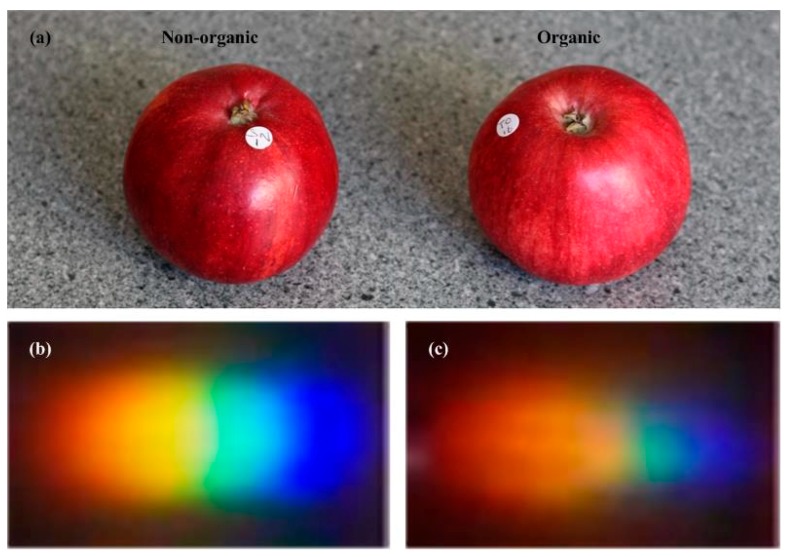
(**a**) Non-organic (**left**) and organic apple (**right**); (**b**) The rainbow image of an organic apple; (**c**) The rainbow image of a non-organic apple.

Figure 5 shows two apples from different classes (organic vs. non-organic) and their rainbow images. Basically, the two apples cannot be visually identified by their physical appearance. If we compare the extracted rainbow images, it is still difficult to tell the difference between the organic and non-organic apples merely based on the radiance or the rainbow size, because specific distinctions may be caused by instrumental and experimental artifacts. In order to differentiate organic apples from non-organic ones precisely, we convert each rainbow image into a sample vector for further analysis.

### 2.4. Feature Vector Representation

Here, we convert the obtained rainbow images to feature vectors in red, green, and blue (RGB) color space in order for representing color information. The RGB color space generally uses the superimpositions of three primary colors to form a diversity of colors. Specifically, it can be represented as a unit cube in 3-dimentional Cartesian coordinate system, where colors are linearly combined by three primary colors of different ratios [23]. This can be represented as:

(8) F=W1·R+W2·G+W3·B,

where F is a certain color, W1, W2 and W3 is the ratio of red, green and blue color luminance, respectively. In this work, we set the value of W1, W2 and W3, respectively to 0.2, 0.7 and 0.1 by trial and error.

**Figure 6:**
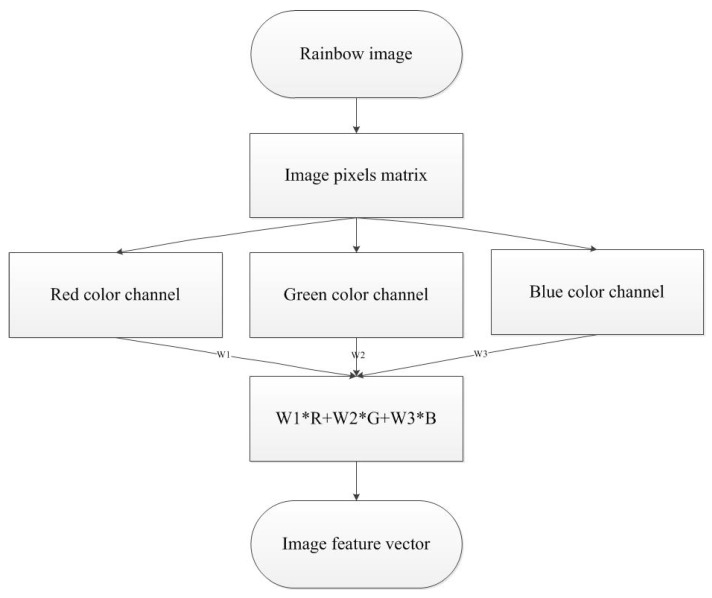
The framework of converting the rainbow image into the image feature vector in RGB color histogram.

**Figure 7:**
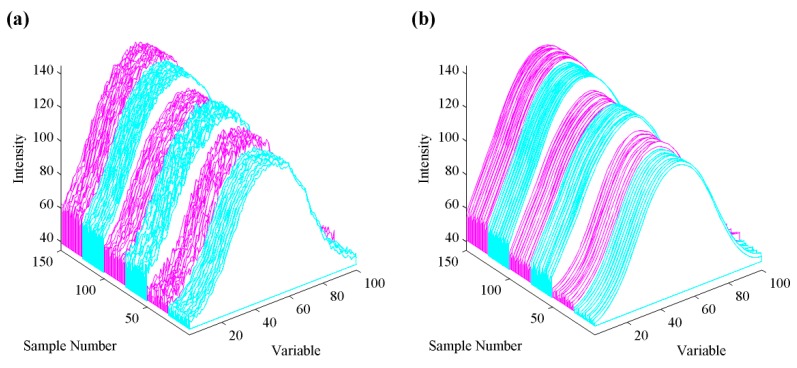
The raw (**a**) and pre-processed (**b**) apple data. Data in cyan and magenta color represents non-organic and organic samples, respectively. Sample 1–50, 51–100 and 101–150 belongs to Gala, Pink lady and Braeburn species respectively.

As each rainbow image (100 by 100 pixels) is comprised of RGB color channels, three image matrices can be transformed into a data matrix according to (8). We calculate the mean of each row and obtain a 1-by-100 feature vector. The framework of converting rainbow image into feature vectors and the obtained raw data are shown in Figure 6 and Figure 7a, respectively.

## 3. Data Analysis

### 3.1. The Nonlinear Problem

**Figure 8:**
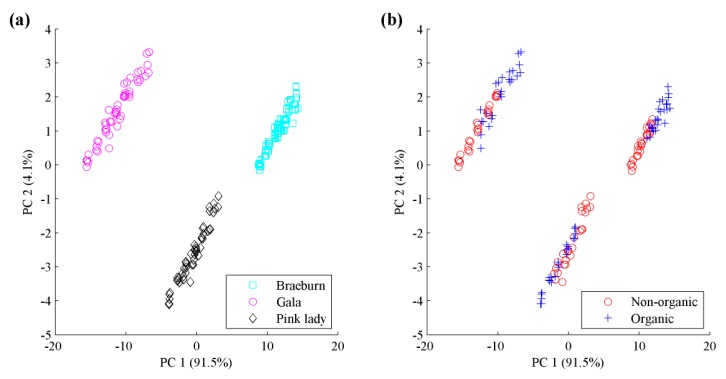
Score plots of the first two dimensions of PCA of apple data: (**a**) Braeburn, Gala and Pink lady species; (**b**) Non-organic and organic types.

From Figure 7a, samples from different apple species present an intuitive distinction within variables ranging from 40 to 60, which shows the three species can be visually identified via the above procedures. However, if we aim to classify these samples as organic or non-organic, this figure can barely provide enough distinctions and the classification decision requires further data analysis. To obtain an overview of the distinctions of species and types, raw apple data were subjected to principal component analysis (PCA), as shown in Figure 8. Different species of apple are well separated while organic and non-organic samples are nonlinearly distributed. We attempt to discover high-performance classification rules for differentiating organic apples from non-organic ones by using a pattern recognition framework.

### 3.2. Pattern Recognition Framework

**Figure 9:**
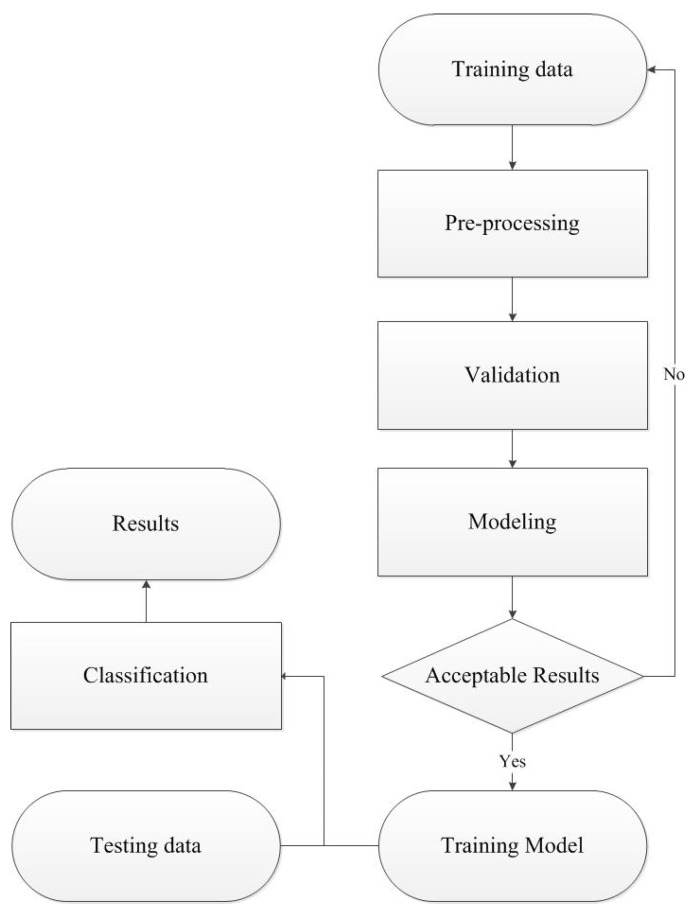
A pattern recognition framework for classifying organic and non-organic apple data.

The pattern recognition framework for the classification of organic and non-organic apple data consists mainly of pre-processing, modelling, validation and classification procedures, as shown in Figure 9. It firstly applies pre-process techniques to reduce noise effects and unwanted variations existed in raw data which are caused by instrumental and experimental artifacts. A suitable pre-processing can decrease the error rate and complexity of classification models. Detailed information about pre-processing apple data will be provided in Section 4. Modelling procedure then uses classification algorithms to reveal the relationship between training data and their corresponding classes. To achieve the highest classification accuracy possible, the parameter(s) of a classifier requires to be optimized in validation procedure. Finally, the optimal model is selected to classify testing data.

### 3.3. Classifiers

Five classification algorithms, namely, *k*-NN, SVM, PLS-DA, KPLS-DA and LW-PLS classifier (LW-PLSC) were applied to classify raw and pre-processed apple data. Among these algorithms, *k*-NN and PLS-DA are baseline methods commonly used in pattern recognition and chemometrics which yield simple and effective models and are computation efficient. SVM, KPLS-DA and LW-PLSC are comparably complex in modelling and generally provide better prediction performance under nonlinear conditions. These algorithms are summarized in the following sections.

#### 3.3.1. *k*-Nearest Neighbors (*k*-NN)

The *k*-NN classification algorithm is a popular method which classifies a query depending on the classes of its neighboring samples. If most of the *k* closest samples belongs to a certain class, the query will be assigned to this class. Specifically, *k*-NN directly assigns the query to the class of its nearest neighbor when *k* equals to 1. The *k*-NN method is theoretically simple but will degrade in performance under high-dimensional condition if the metric is based on Euclidean distance [24].

#### 3.3.2. Support Vector Machine (SVM)

The SVM classifier aims to find an optimal hyperplane which correctly separates the samples of the different classes while maximizing the shortest distances from the hyperplane to the nearest samples for each class [25]. It can be extended to nonlinear classification by mapping the input data into feature space via kernel functions.

#### 3.3.3. Partial Least Squares Discriminant Analysis (PLS-DA)

PLS regression is a standard method for processing chemical data which assumes the investigated system is driven by a set of underlying latent variables (LVs, also called latent vectors, score vectors, or components). It extracts LVs by projecting both ***X*** and ***Y*** onto a subspace such that the pairwise covariance between the LVs of ***X*** and ***Y*** is maximized. To ensure the mutual orthogonality of the LVs, this procedure is iteratively carried out by using deflation scheme which subtracts from ***X*** and ***Y*** the information explained by their rank-one approximations based on score vectors. PLS-DA is the classification use of PLS regression by transforming categorical responses into numerical responses using dummy matrix coding [26]. 

#### 3.3.4. Kernel Partial Least Squares Discriminant Analysis (KPLS-DA)

KPLS-DA maps the original data ***X*** into Hilbert feature space ***F*** (*φ*: ***R****^d^* → ***F***):

***K****_ij_* = *k* (***x****_i_*, ***x_j_***) = (*φ* (***x****_i_*), *φ* (***x****j*)) = *φ* (***x_i_***)^T^*φ* (***x****_j_*) (9)

and then constructs a PLS-DA model for classification. The mapping procedure is performed by kernel function which calculates the similarity between two sample vectors. In this paper, we use the Gaussian kernel due to its efficiency which is defined as:

(10) $$K_{ij}=e^{-\frac{{\left|\left|x_{i}-x_{j}\right|\right|}^{2}}{2\sigma^{2}}},$$

where *σ* is Gaussian width of the kernel function.

#### 3.3.5. Locally Weighted Partial Least Squares Classifier (LW-PLSC)

LW-PLSC is a JIT method proposed in our recent work for modelling nonlinear data, which extends LW-PLS to the classification use. For a given query, it respectively enlarges and lessens the influence of neighboring and remote samples towards a PLS-DA model. As a result, the global nonlinearity can be lessened. Two parameters of LW-PLS, localization parameter *φ* and LVs controls sample weights and model complexity, respectively.

### 3.4. Performance Evaluation

We firstly partition the apple data into training and testing sets by using DUPLEX splitting [27] with the ratio of 2:1. DUPLEX splitting maintains the same diversity in both sets, so that the data in each set follows the statistical distribution of the overall data [28]. Then leave-one-out cross validation is implemented to obtain the optimal parameter(s) of each algorithm and the validation accuracy. With the selected optimal parameter(s), each algorithm constructs a classification model on training set and use it to predict samples from testing set. The classification results are finally evaluated by overall accuracy and per-class accuracy.

## 4. Results and Discussion

### 4.1. Data Pre-Processing

As the image data collected by our sensor system is a new type of data, to the best of our knowledge, there is no report in the literature about how to pre-process such data. We adopt typical pre-processing techniques used in spectroscopy and signal processing to improve the classification performance as well as the simplicity of models, including smoothing, baseline correction and normalization. The basic requirement of pre-processing is that it should decrease or maintain the model complexity without significant loss of useful information. By checking these techniques and their combinations, only Savitzky-Golay smoothing [29] (fitted by a polynomial of degree two and a 33-point moving window) can best improve the validation performance of five algorithms. Figure 7b shows the effects of smoothed data. The validation accuracy of smoothed data drastically exceeds that of raw data, as shown in Table 1.

### 4.2. Parameter Optimization and Classification Performance

The optimal parameters of five algorithms are set by leave-one-out cross validation on training set. The number of nearest neighbors in *k*-NN is chosen from 1 to 49 with an interval of 2, while the number of LVs in PLS-based methods does not exceed 10 in case of overfitting. We set the value of penalty parameter in SVM from 1 to 8 and select Pearson VII kernel function (PUK). A grid search approach is used in KPLS-DA (LV * s) and LW-PLSC (LV * *φ*). The *σ* in KPLS-DA is adjusting from 10^−3^ to 10^3^ on a logarithmic scale, while the *φ* in LW-PLSC is varying from 0.1 to 25.

**Figure 10:**
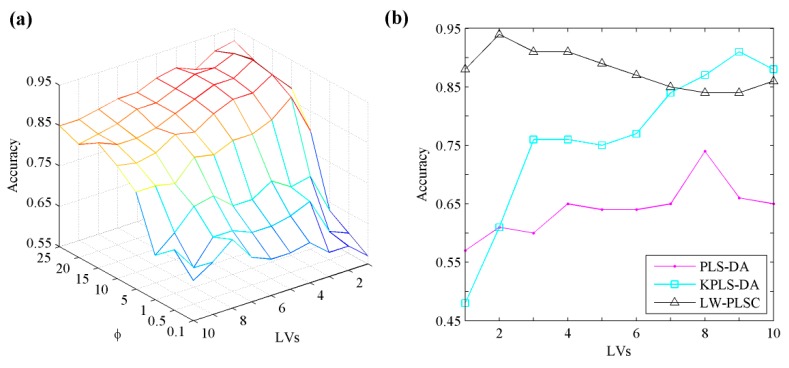
The accuracy of leave-one-out cross validation of LW-PLSC with varying parameters (**a**) and the performance of three PLS-based algorithms (**b**) on apple data.

Here, we demonstrate the grid search of the optimal number of LVs and *φ* for LW-PLSC, which is depicted in Figure 10a, as a mesh plot. LW-PLSC obtains the peak validation accuracy when the number of LVs and *φ* equals to 2 and 15, respectively. We further graphically present three PLS-based algorithms with varying LVs in Figure 10b by fixing other parameters (*σ* in KPLS-DA and *φ* in LW-PLSC) to their optimal values. LW-PLSC drastically outperforms PLS-DA for each LV and achieves the highest accuracy of 94% by selecting only 2 LVs. This demonstrates locally weighted modelling has low complexity and is efficient in capturing the data nonlinearity. KPLS-DA can also provide a comparable result to LW-PLSC but uses 7 more LVs. The validation results of five algorithms and their corresponding optimal parameters are provided in Table 1. Both *k*-NN and SVM obtain around 90% validation accuracy.

The overall classification accuracy and per-class accuracy on testing set are shown in Table 1. LW-PLSC achieves the highest overall accuracy of 94% among five algorithms, while SVM and KPLS-DA present the same performance at 92%. PLS-DA yields the least precise result due to the nonlinear distribution of data. By looking at the classification accuracy of each class, SVM achieves the best result of 92.7% for identifying the non-organic class, whereas KPLS-DA achieves the best result of 100% for identifying the organic class. LW-PLC obtains comparably balanced accuracies for every class so it achieves the best overall accuracy of 94%.

## 5. Conclusions

This paper presents a new sensor system, which consists of low-cost hardware and pattern recognition software, for food authentication, i.e., differentiating organic apples from non-organic ones. The sensor system can effectively acquire rainbow images from food samples, which are converted into non-standard spectral data. To overcome the instrumental and experimental artifacts in such non-standard spectral data and to address the inherent nonlinearity problem in such data, appropriate and effective pre-processing and locally weighted modelling are adopted. Experiments show that the proposed sensor system has achieved a highest classification accuracy of 94% for identifying and distinguishing between organic and non-organic apples, demonstrating the potential of the new sensor system as a rapid, non-destructive and low-cost solution for food authentication. In our future work, we will optimize the hardware components and test the sensor system on a variety of foods.

## Figures and Tables

**Table 1:** The overall and per class classification accuracy (%) of the different algorithms on apple data.

	Training		Testing			Parameters	
	Raw	Pre-Processed	Overall	Non-Organic	Organic		
*k*-NN	72	91	84	75	90	NN: 1	
SVM	80	89	92	92.7	86.7	C: 4	Kernel: PUK
PLS-DA	70	74	52	36.4	64.3	LVs: 8	
KPLS-DA	88	91	92	81.8	100	LVs: 9	*σ*: 100
LW-PLSC	87	94	94	89.5	96.8	LVs: 2	*φ*: 15
